# Rational design and *in vivo* selection of SHIVs encoding transmitted/founder subtype C HIV-1 envelopes

**DOI:** 10.1371/journal.ppat.1007632

**Published:** 2019-04-03

**Authors:** Sean P. O’Brien, Adrienne E. Swanstrom, Amarendra Pegu, Sung-Youl Ko, Taina T. Immonen, Gregory Q. Del Prete, Christine M. Fennessey, Jason Gorman, Kathryn E. Foulds, Stephen D. Schmidt, Nicole Doria-Rose, Carolyn Williamson, Theodora Hatziioannou, Paul D. Bieniasz, Hui Li, George M. Shaw, John R. Mascola, Richard A. Koup, Peter D. Kwong, Jeffrey D. Lifson, Mario Roederer, Brandon F. Keele

**Affiliations:** 1 AIDS and Cancer Virus Program, Frederick National Laboratory for Cancer Research, Frederick, MD, United States of America; 2 Vaccine Research Center, National Institute of Allergy and Infectious Diseases, National Institutes of Health, Bethesda, MD, United States of America; 3 Division of Medical Virology, University of Cape Town, Cape Town, South Africa; 4 Centre for the AIDS Programme of Research in South Africa, University of KwaZulu-Natal, Durban, South Africa; 5 The Rockefeller University, New York, NY, United States of America; 6 Howard Hughes Medical Institute, The Rockefeller University, New York, NY, United States of America; 7 Department of Medicine, University of Pennsylvania, Philadelphia, PA, United States of America; Emory University, UNITED STATES

## Abstract

Chimeric Simian-Human Immunodeficiency Viruses (SHIVs) are an important tool for evaluating anti-HIV Env interventions in nonhuman primate (NHP) models. However, most unadapted SHIVs do not replicate well *in vivo* limiting their utility. Furthermore, adaptation *in vivo* often negatively impacts fundamental properties of the Env, including neutralization profiles. Transmitted/founder (T/F) viruses are particularly important to study since they represent viruses that initiated primary HIV-1 infections and may have unique attributes. Here we combined *in vivo* competition and rational design to develop novel subtype C SHIVs containing T/F envelopes. We successfully generated 19 new, infectious subtype C SHIVs, which were tested in multiple combinatorial pools in Indian-origin rhesus macaques. Infected animals attained peak viremia within 5 weeks ranging from 10^3^ to 10^7^ vRNA copies/mL. Sequence analysis during primary infection revealed 7 different SHIVs replicating in 8 productively infected animals with certain clones prominent in each animal. We then generated 5 variants each of 6 SHIV clones (3 that predominated and 3 undetectable after pooled *in vivo* inoculations), converting a serine at Env375 to methionine, tyrosine, histidine, tryptophan or phenylalanine. Overall, most Env375 mutants replicated better *in vitro* and *in vivo* than wild type with both higher and earlier peak viremia. In 4 of these SHIV clones (with and without Env375 mutations) we also created mutations at position 281 to include serine, alanine, valine, or threonine. Some Env281 mutations imparted *in vitro* replication dynamics similar to mutations at 375; however, clones with both mutations did not exhibit incremental benefit. Therefore, we identified unique subtype C T/F SHIVs that replicate in rhesus macaques with improved acute phase replication kinetics without altering phenotype. *In vivo* competition and rational design can produce functional SHIVs with globally relevant HIV-1 Envs to add to the growing number of SHIV clones for HIV-1 research in NHPs.

## Author summary

Nonhuman primates provide useful models for studying HIV transmission, pathogenesis and cure strategies. Due to species-specific antiviral factors, however, HIV cannot replicate in Asian macaques directly. Some chimeric viruses incorporating HIV Envelope genes in simian immunodeficiency virus (SIV) backbone can replicate to sufficient levels in Asian macaques to permit evaluation of anti-HIV interventions. Here we describe the generation of new SHIV clones unique to the field in 4 important ways. First, these clones were generated from the globally relevant HIV-1 subtype C, which is the most prevalent form of HIV globally and is found predominately in sub-Saharan Africa where the pandemic is particularly devastating but is poorly represented among SHIVs studied to date. Second, we utilized Envelope genes from viruses that established primary infection, making these clones particularly useful in transmission studies. Third, these clones were not generated by animal passage, which may alter some of the unique properties of these Envelopes. Finally, we used direct within animal competition studies and two targeted mutations to select highly replicative clones. We provide here both the discovery of new SHIV clones, and also a process to generate additional clones in the future.

## Introduction

Asian macaques infected with simian immunodeficiency virus (SIV) are commonly used to model HIV-1 infection in humans. This model has become the most widely accepted animal models for HIV/AIDS research due to its ability to accurately recapitulate key aspects of human HIV infection, including rapid turnover and progressive depletion of mucosal and peripheral CD4^+^ T cells, sustained high viral load, chronic immune activation, progressive immunodeficiency, and the eventual onset of life-threatening opportunistic infections and tumors [1].

Passive transfer of SIV neutralizing antibodies (NAbs) conferred protection to macaques from SIV infection [2–6], suggesting that immunological protection against HIV-1 is possible. However, the Env glycoproteins, to which neutralizing antibodies are exclusively directed [7, 8], are sufficiently different between SIV and HIV-1 to make SIV models not useful for directly testing human antibody-based therapies against HIV-1 [9–11]. Low-level replication following HIV-1 inoculation has been demonstrated in *Macaca nemestrina* (pig-tailed macaques) [12, 13] likely due at least in part to the lack of TRIM5α mediated restriction in this species, but rhesus macaque restriction factors like APOBEC3, Tetherin, SAMHD1, and TRIM5α limit HIV-1 replication [12, 14–16], and the resulting levels of viral replication are too low and too transient to allow testing of therapeutic interventions [17].

To address this situation, investigators have sought to develop chimeric viruses that incorporate HIV-1 Env but can replicate well in macaques. Designated Simian-Human Immunodeficiency Viruses (SHIVs), these viruses comprise an SIV genome with HIV-1 *tat*, *rev*, *vpu*, and *env* replacing the SIV *tat*, *rev*, and *env* genes [18]. SHIV models in macaques have been used to demonstrate the efficacy of passive transfer of HIV-1 Nabs to prevent viral transmission [19–22] although they often do not replicate as well as SIV in NHPs, particularly in the chronic phase of infection [23–31]. With improved methods to isolate NAbs [8], SHIV models continue to be used extensively for passive antibody infusion studies [32–34] and for preclinical evaluation of Env targeted vaccines [35].

Most SHIVs, however, show low peak viremia with limited, if any, chronic phase viral replication and associated pathogenesis [23–31] likely at least in part due to the suboptimal interaction of most HIV-1 Envs with macaque CD4 [36]. Serial passage through macaques enables SHIVs to adapt to the NHP host, which can result in higher peak viremia and persistence and, in some cases, progression to AIDS defining endpoints such as with SHIV SF162P3 and SHIVAD8.EO [23, 26, 27, 37–39]. However, this approach has pitfalls. Neutralization sensitivity of the HIV-1 Env may change as a result of *in vivo* adaptation since the Env trimer may adopt a more open conformation to enhance binding to macaque CD4 [27, 34, 37, 40–42]. Alterations in quaternary structure can make Env resistant to antibodies like PG9, PG16, PGT145, and VRC03 that recognize quaternary epitopes while simultaneously rendering the virus more sensitive to antibodies like Mab 17b that recognize CD4-induced epitopes [43]. Additionally, tropism may shift from the CCR5 co-receptor that is relevant in initial infection to the CXCR4 co-receptor that can be utilized more frequently in chronic phase infection [27]. Transmitter/Founder (T/F) viruses are almost exclusively CCR5 tropic with CXCR4 or X4/R5 dual-tropic virus developing later in infection. SHIV89.6P, a SHIV generated by serial passage in macaques that was used frequently because it consistently showed a high level of early replication, rapid CD4^+^ lymphocyte depletion, and fast progression to an AIDS-like illness in macaques, provides a cautionary tale [24, 37]. While the parental HIV-1 89.6 Env in SHIV89.6 was nominally X4/R5 dual tropic *in vitro*, serial passage leading to SHIV89.6P selected for a virus that behaved as an exclusively CXCR4 -tropic virus *in vivo*, not reflective of the vast majority of transmitted HIV-1s. This led to an altered mode of pathogenesis, distinct from that of HIV-1, characterized by the near complete early depletion of naïve CD4^+^ T cells thus undermining the development of antigen specific CD4^+^ T cell help and masking its unusual sensitivity to autologous NAbs. Thus, intervention strategies may appear efficacious when they are not [44, 45]. CXCR4 tropic viruses are no longer considered appropriate challenge viruses for evaluating early HIV-1 intervention strategies.

In rare cases some SHIVs do not require passage to replicate robustly in macaques [28–30, 46], and these Envs maintain native quaternary epitopes and CCR5 tropism [43]. However, screening for them is time and resource intensive and does not allow for prospectively designed studies where analyses of particular HIV-1 Envs in the context SHIV infection is desired (e.g., SHIVs bearing HIV-1 Envs that in humans elicited broadly neutralizing antibodies or non-neutralizing ADCC antibodies). To develop new NHP models that better recapitulate features of HIV-1 infection in humans, novel rational design strategies are needed to engineer T/F HIV-1 Envs of interest to produce SHIVs that replicate robustly *in vivo* with clinically relevant replication kinetics and neutralization profiles.

To enhance SHIV fitness in macaques, several recent studies investigated Env point mutations that improve macaque CD4 binding. Humes *et al* identified two residues in HIV-1 Env (at sites 312 and 204—HxB2 numbering), which, when mutated, increased infectivity in pig-tailed macaque cells *in vitro* [47]. While tropism was not altered, neutralization sensitivity increased for antibodies targeting CD4-induced epitopes and decreased for antibodies targeting quaternary epitopes [43, 47]. Recently, Li *et al* reported enhanced Env affinity for rhesus CD4 without an appreciable impact on tropism or antibody mediated neutralization sensitivity when bulky hydrophobic or basic amino acids (methionine, tyrosine, histidine, tryptophan, or phenylalanine) were substituted for serine at residue 375 in Env [31]. Notably, rhesus macaques inoculated with SHIVs bearing Env375 variants all became productively infected with acute viral loads comparable to humans with HIV-1. Additionally, Del Prete *et al* reported a naturally arising threonine/valine substitution for alanine at residue 281 during *in vivo* adaptation [38]. This mutation enhances HIV-1 Env binding to macaque CD4, resulting in improved replication in macaque cells without adversely affecting Env neutralization sensitivity.

In this study we investigated the different emerging solutions for engineering SHIVs with increased replication in macaques, identifying functional Envs by a combination of screening and rational design at residues 375 and 281. We evaluated 20 different T/F subtype C Env clones derived from South African study participants soon after transmission [48]. 19 of 20 Env clones were successfully cloned into a full-length SIV backbone as a replication competent SHIV. These Envs represent a wide diversity in neutralization sensitivity and as SHIV clones could facilitate preclinical studies using authentic T/F Envs from a globally important subtype. Using a pooled inoculum *in vivo* screening approach, 7 SHIV clones that replicated well in macaques were identified. From these, we selected the 3 most successful SHIVs and 3 SHIVs that were not detected in the plasma of any animal after pooled inoculations and introduced mutations at Env residue 281 and/or 375 to assess whether viral replication could be improved. Mutants at Env375 replicated well *in vitro* and were found at high proportions both at peak viremia and in the chronic phase of infection after inoculation of macaques. Mutation at Env281 likewise improved replication *in vitro*; however, some Env281 variants exhibited compromised infectivity. Mutations at 281 or 375 did not change neutralization sensitivity and combining mutations did not lead to any incremental improvement in replication. Thus, screening and genetic engineering can produce SHIVs capable of robust *in vivo* replication with preserved neutralization profiles, without the requirement for further *in vivo* adaptation and Env evolution. Furthermore, mutation can enable previously non-replicating SHIVs to replicate in macaques, thus expanding the repertoire beyond rare, naturally occurring Envs that can inherently bind rhesus CD4 well.

## Results

### *In vivo* replication and selection of T/F subtype C SHIV

**Fig 1:**
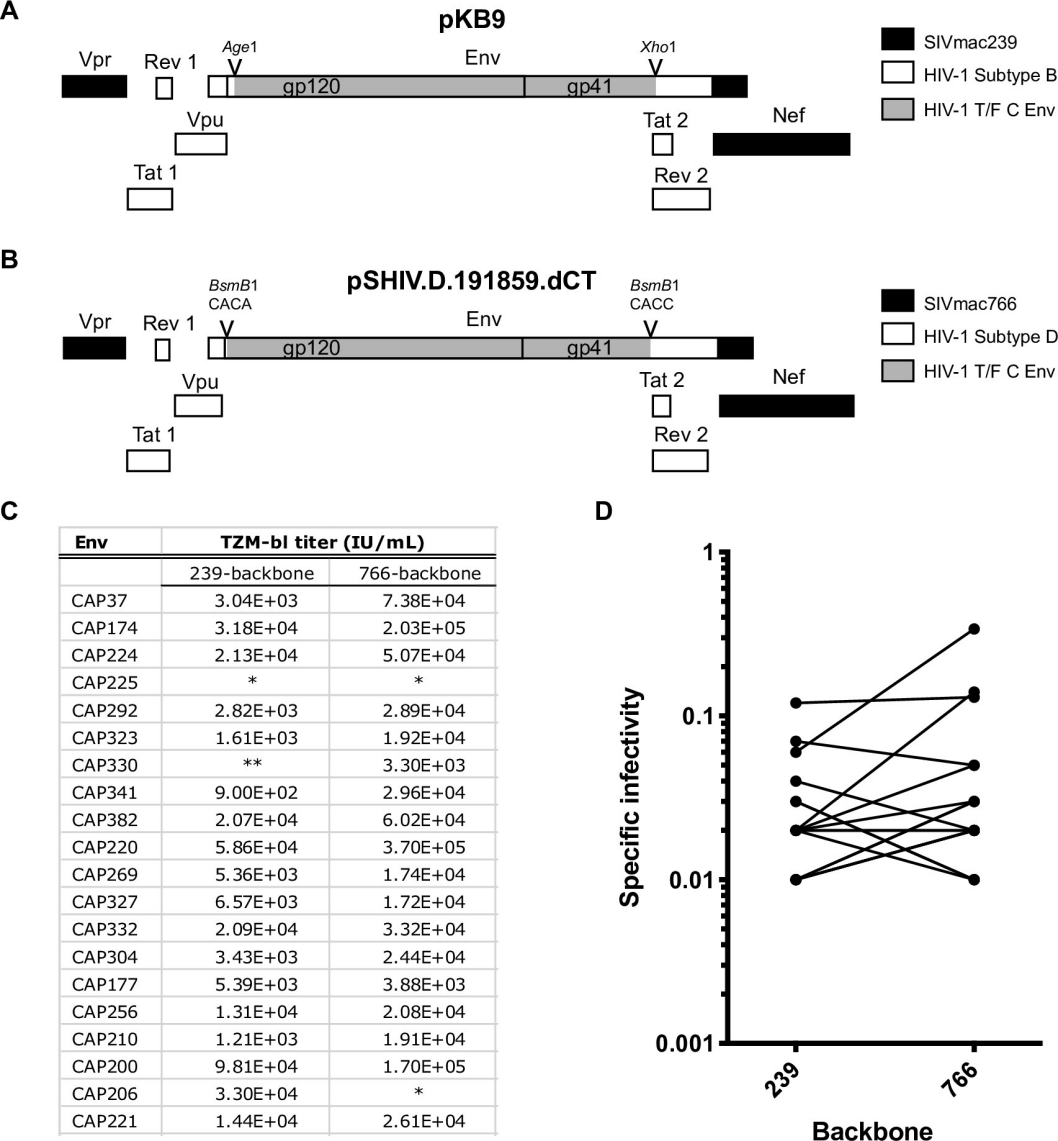
Subtype C SHIV cloning and infectivity titration. Schematic representation of a portion of the 3’ viral genome for SIVmac239-based KB9 clones (A) and SIVmac766-based SHIV.D.191859.dCT clones (B). We introduced 19 HIV-1 T/F subtype C *env* sequences to replace the native *env* sequence after the signal peptide in both backbones using the internal *Age*1 and *Xho*1 restriction sites (A) or the Type IIS restriction enzyme recognition site *BsmB*1 (with digestion sequence shown) within *env* to permit seamless cloning of various HIV-1 *envs* (B). TZM-bl titers for each HIV-1 *env* in SIVmac239- or SIVmac766-backbone (C). An * indicates a failure to clone, while ** indicates a lack of infectivity on TZM-bl cells. Specific infectivity (TZM-bl titer normalized to p27 gag) of each functional SHIV in the SIVmac239- and SIVmac766-backbone (D).

20 T/F subtype C *env* sequences derived from acutely HIV-1 infected individuals from South Africa [48] representing a wide range of subtype C viral diversity (S1 Fig) were subcloned into both the pKB9 vector [37] and in pSHIV.D.191859.dCT vector [31](Fig 1A and 1B). Because the KB9 backbone is composed of the chronically passaged SIVmac239 (*gag*, *pol*, *vif*, *vpx*, and *vpr*) and genes from a chronic subtype B HIV-1 strain HXBc2 (*tat*, *rev*, *vpu*, and *env*) [24] it may not be representative of authentic T/F virus. We wanted to directly compare the performance of 239-based SHIVs to the newly described pSHIV.D.191859.dCT [31], which contains *tat*, *rev*, *vpu*, and *env* genes from a T/F subtype D HIV-1 strain introduced into the T/F SIVmac766 genome (a derivative of SIVmac251). We successfully cloned 19 out of the 20 HIV Envs into at least one of the two SIV backbones, 17 of which were successfully cloned into both SHIV platforms (Fig 1C). The specific infectivity of these Env matched clones was determined using the TZM-bl titer normalized to p27 gag (pg/ml) which indicated that infectivity was not significantly different between backbones (p = 0.3, Wilcoxon signed-rank test) (Fig 1D).

**Fig 2:**
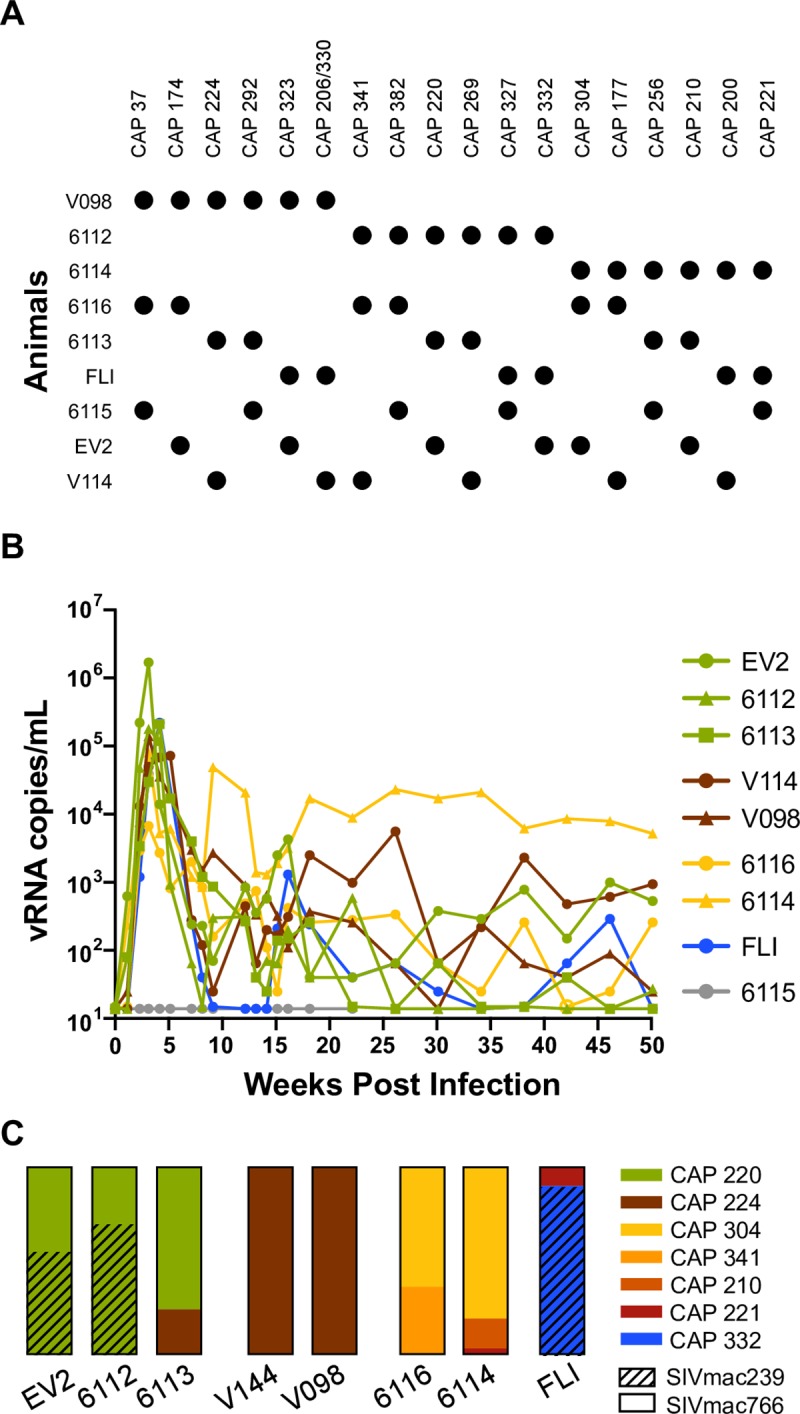
Pooled inoculation strategy and *in vivo* replication of subtype C SHIVs. (A) Nineteen unique SHIVs were grouped into 9 pools with 6 variants in each pool. Each variant was represented in 3 pools, and pairs did not occur more than twice. Virus inocula contained SHIVs based on either the SIVmac239 or SIVmac766 backbone in equal proportion except for CAP206_239_ and CAP330_766_ for which only one backbone was available. Infectious titer determined on TZM-bl cells for each SHIV was used to generate virus pools containing equal amounts of each constituent SHIV. (B) Plasma viral load in 9 rhesus macaques intravenously inoculated with the pooled SHIVs. Infectious titer determined on TZM-bl cells for each SHIV was used to generate virus pools containing equal amounts of each constituent SHIV. (C) *In vivo* proportions of each clone at peak viremia in plasma were assessed by sequence analysis and are represented by stacked bar graphs. SHIVs with SIVmac766 backbone are shown in solid and those with a SIVmac239 backbone are shaded. Colors in VL graph correspond to the clone of highest frequency in that animal.

To identify SHIVs with robust *in vivo* replication, 9 rhesus macaques were infected intravenously using a combinatorial pool infection strategy (Fig 2A). SHIV clones were divided into 9 partially overlapping pools with 6 envelopes represented in each pool. Each envelope appeared 3 times across pools, and pairs within pools did not occur more than twice. Each pool was inoculated into one naïve rhesus macaque. Virus challenge stocks contained equal proportions of the SIVmac239 and SIVmac766 backbone for each of the Env clones with the exception of CAP206 and CAP330, since each had only one backbone (SIVmac239 and SIVmac766 respectively) for a total of 36 viruses.

By using this strategy of *in vivo* competition, we identified viruses containing Env sequences that confer the highest comparative fitness *in vivo* while minimizing the number of animals. Of the 9 animals infected with the virus pools, 8 showed evidence of productive infection with peak viremia occurring between 21 to 35 days post infection (dpi) and peak viral load ranging from 6.7×10^3^ to 1.7×10^6^ vRNA copies/mL (Fig 2B). For wildtype SIVmac239 infection of rhesus macaques, peak viremia typically ranges between 10^7^−10^8^ vRNA copies/mL and occurs between 10 and 17 dpi followed by a chronic phase viral load between 10^5^−10^7^ vRNA copies/mL [1]. Only animal 6114 exhibited a steady, chronic phase viral load greater than 10^3^ vRNA copies/mL. At the end of 50 weeks, 6 animals had detectable viremia between 25 and 5,200 copies/mL. CD4^+^ T cell depletion was not observed during peak viremia nor during the chronic phase of infection (S2 Fig). The proportion of each inoculated virus clone in plasma at peak viremia was assessed by sequence analysis (Fig 2C). We found 4 Envs (CAP220, CAP224, CAP304, and CAP332) at much higher proportion than the other 15 SHIVs present in the inoculum pools. In 2 animals (EV2 and 6112) only CAP220 was detected at peak viremia with roughly equal proportion of the 2 different backbones. In animal 6113, CAP220 was the major clone with CAP224 also detected, but both were found exclusively in the SIVmac766 backbone. For animals V144 and V098, CAP224 was found exclusively, and again the successful clone was found only in the context of the SIVmac766 backbone. In 2 other animals (6116 and 6114) CAP304 was the dominant clone, with CAP341 as the minor lineage in animal 6116, and CAP210 and CAP221 as minor lineages in animal 6114. The SIVmac766 backbone was found in all detectable clones in both 6116 and 6114. Finally, in animal FLI, 2 clones were identified: CAP332 (SIVmac239 backbone) and the minor lineage CAP221 (SIVmac766 backbone). When comparing the relative frequency of each backbone, it was determined that the SIVmac766 backbone was significantly more abundant *in vivo* compared to the SIVmac239 backbone (p = 0.01, Wilcoxon signed-rank test).

### *In vitro* replication of Env375 mutated SHIVs

**Fig 3:**
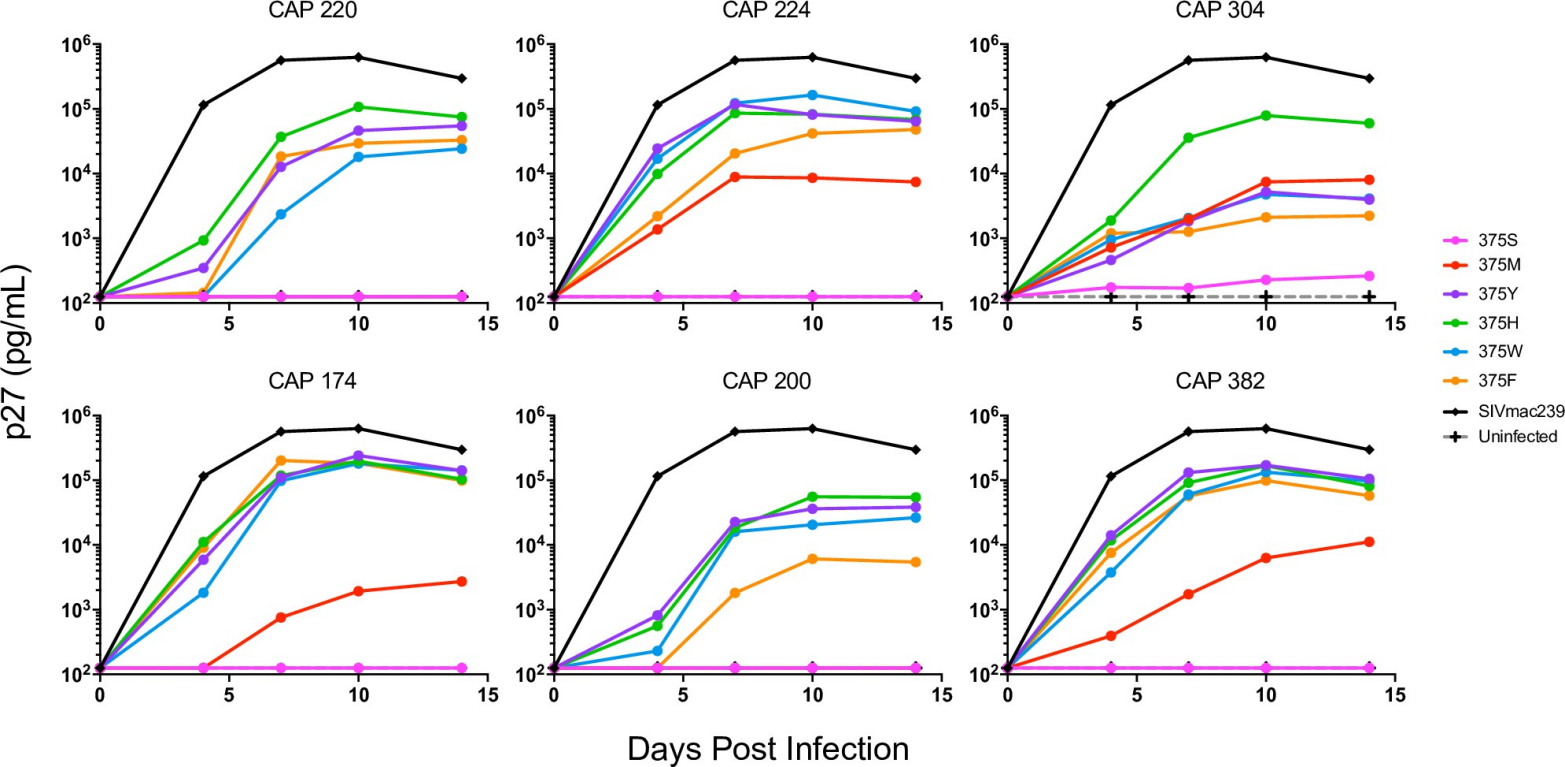
*In vitro* replication of SHIV 375 mutants. *In vitro* replication of each Env375 polymorphism was assessed by measuring p27 levels in activated primary rhesus CD4^+^ T cells over the course of 14 days. Individual SHIVs were used to infect 10^6^ activated rhesus CD4^+^ T cells at a nominal MOI of 0.02. Culture supernatants were collected over 14 days, with media replacement at each collection time-point to maintain cultures in 2 mL total volume. Viral p27 protein was quantified by ELISA. Each polymorphism is represented by a different color.

Strategies to increase SHIV replication in macaques by rational design rather than adaptation through serial passage in animals provide the opportunity to create more highly replicative clones while minimizing the risk of unintended alterations of viral phenotype. Thus, to determine the effects of mutations at Env375 on virus replication we introduced this mutation in two types of viruses: 1) our *in vivo*-selected SHIVs (CAP220_766_, CAP224_766_, and CAP304_766_) that were able to replicate efficiently *in vivo* without modification and 2) SHIVs (CAP174_766_, CAP200_766_, and CAP382_766_) that were not detected in any animal during our initial *in vivo* screen, despite having been present at the same level in the pooled inoculum. For each of the 6 SHIV clones, we mutated the serine (S) in the Env375 position to methionine (M), tyrosine (Y), histidine (H), tryptophan (W), or phenylalanine (F), all in the context of the SIVmac766 backbone. Replication was then measured following *in vitro* infection of naïve primary rhesus CD4^+^ T cells (Fig 3). Each polymorphism was assessed individually on the same donor cells by measuring p27 levels over a 14-day period. For each Env, virus with the wildtype 375S replicated either poorly or was not detectable above background. Mutants with 375M also failed to replicate (CAP200_766_ and CAP220_766_) or replicated to low levels in CAP174_766_, but were marginally better in CAP224_766_, CAP304_766_ and CAP382_766_. By contrast, the other Env375 mutations (Y, H, W, F) increased replication in these subtype C SHIVs. Overall these data are consistent with Li *et al*, who showed increased viral replication with 375M and 375S mutations in subtype B clones and 375Y, H, W and F for subtype C SHIVs [31].

### In vivo replication of Env375 mutated SHIVs

**Fig 4:**
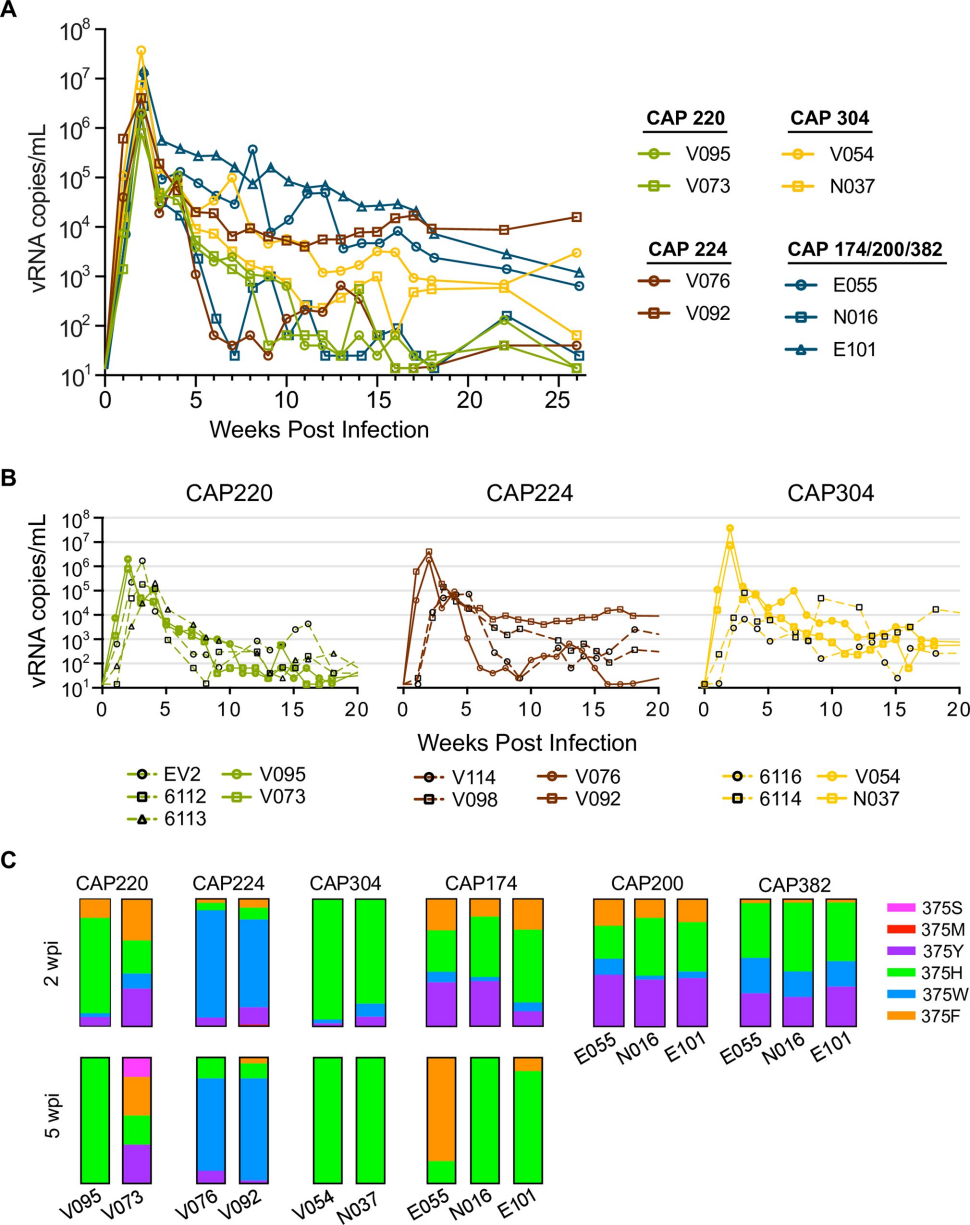
*In vivo* replication of SHIV 375 mutants. (A) Plasma viral load in 9 rhesus macaques intravenously inoculated with Env375 viral variants. Pairs of rhesus macaques were each inoculated with pooled variants of CAP220, CAP224, or CAP304. All polymorphic variants of the previously undetectable CAP174, CAP200, and CAP382 were pooled to infect 3 rhesus macaques. (B) Comparison of VL for rhesus macaques inoculated with pooled SHIV (dashed lines) that included the Envs indicated and Env375 variants (solid lines) from these clones. (C) Proportions of Env375 variants in blood plasma as determined by Miseq sequence analysis at peak viremia (14 dpi) and by single genome sequencing at chronic infection (35 dpi) are represented by stacked bar graphs.

Next, we challenged 9 animals intravenously with the Env375 mutant clones (Fig 4). For each SHIV clone that outcompeted the other *in vivo* (CAP220_766_, CAP224_766_, and CAP304_766_), all 6 Env375 variants were pooled and used to infect 2 rhesus macaques per SHIV family. The Env375 variants of the 3 SHIVs previously not detected *in vivo* (CAP174_766_, CAP200_766_, and CAP382_766_) were pooled together and used to infect 3 additional rhesus macaques. All animals showed evidence of productive infection with peak viral loads of 7.9×10^5^−3.8×10^7^ vRNA copies/mL at 14 dpi (Fig 4A). This peak viral load was ~1 log higher than animals infected with the original pools with only the wild-type Env375S and was achieved one to two weeks earlier (Fig 4B). Notably, animals infected with the previously undetectable CAP174, CAP200, and CAP382 clones manifested viral replication dynamics similar to those animals infected with CAP220, CAP224, or CAP304 with peak viremia between 2.8×10^6^ and 1.5×10^7^ vRNA copies/mL. Sequence analysis of the entire *env* gene at acute peak viremia revealed CAP174_766_ to be the dominant clone in these animals (97%) with CAP200_766_ at 2% and CAP381_766_ at 1%. Total CD4^+^ T cell counts were measurably reduced at one-week post infection for 7 of the 9 infected animals (S2 Fig). This reduction was transient and returned to baseline during the chronic phase of infection.

Next, sequence analysis was used to determine the proportions of each Env375 mutation within each animal at peak (2 weeks post infection—wpi) and in early chronic phase viremia (5wpi). Clones with histidine, tryptophan, tyrosine, or phenylalanine at Env375 were all found at high proportions at peak viremia across all animals (Fig 4C), as was predicted from our *in vitro* replication experiments. For most SHIVs, clones with histidine consistently constituted the largest proportion of the variants (in all cases greater than 26% and as high as 94% with a median of 46%). In animals infected with CAP224_766_, virus with tryptophan at Env375 made up the largest proportion at 84% and 69% for animals V076 and V092 respectively. Proportions of each Env375 mutation at 5 wpi revealed histidine as the only detectable variant for CAP304_766_ and the dominant variant for CAP220_766_ in V095 (100%) and for CAP174_766_ in N016 (97%) and in E101 (89%). CAP220_766_ in V073 remained heterogeneous for histidine (23%), phenylalanine (31%), and tyrosine (31%), and showed an emergence of parental serine (15%). Phenylalanine became the dominant mutant for CAP174_766_ in E055 (82%). In animals infected with CAP224_766_ variants, clones with tryptophan continued to make up the largest proportion at 73% and 81% for V076 and V092 respectively with histidine, tyrosine, and phenylalanine also being detected. Overall, the most dramatic increases in viral replication due to 375 mutations were seen in those viruses where the original clone performed the worst (e.g. clone CAP174_766_ failed to replicate with wildtype 375S but replicated to high titers with 375 mutations, while viruses that replicated well). For CAP304_766_, we saw a dramatic increase in peak viral load which was less prominent in CAP224_766_ and nonexistent in CAP220_766_. Interestingly, time to peak viral load was earlier in all animals infected with Env375 mutants.

### Replication of Env281 mutated SHIVs

**Fig 5:**
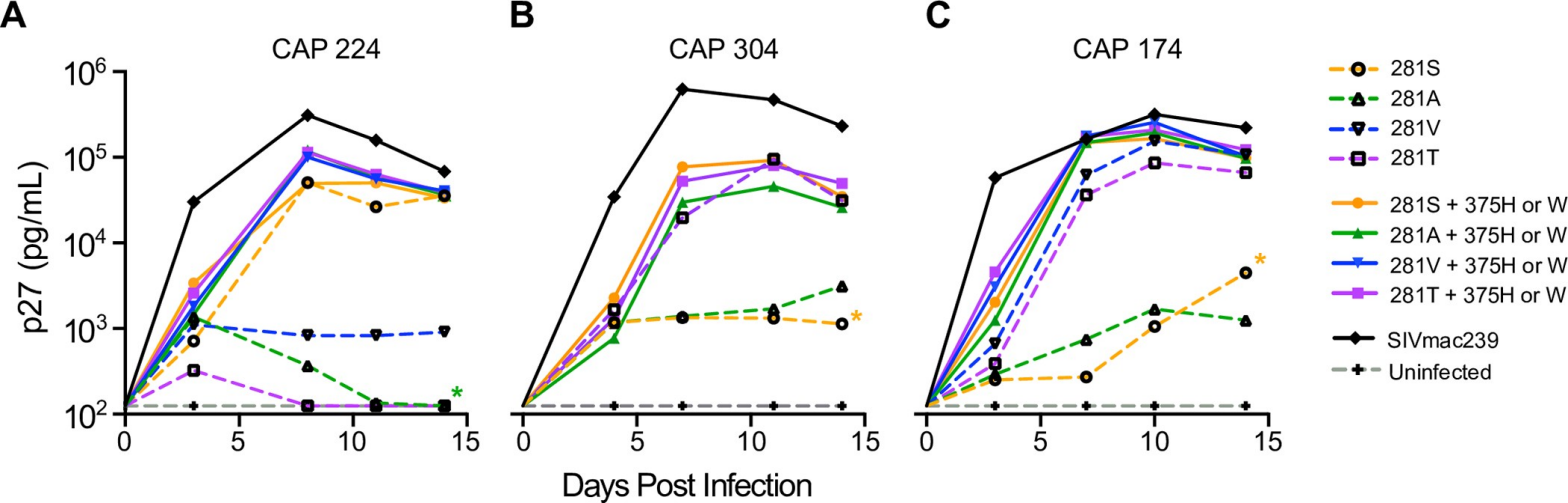
*In vitro* replication of SHIV 281/375 mutants. We tracked *in vitro* replication of each Env281/375 polymorphism for (A) CAP224, (B) CAP304, and (C) CAP174 by measuring p27 levels in activated primary rhesus CD4^+^ T cells over the course of 14 days. Individual SHIVs were used to infect 10^6^ activated rhesus CD4^+^ T cells at a MOI of 0.01. Culture supernatants were collected over 14 days, with media replacement at each collection time-point to maintain cultures in 2 mL total volume. Viral p27 protein was quantified by ELISA. Env375 mutants contain a histidine except for CAP224 which has a tryptophan mutation. Wildtype 281 virus cultures are denoted (*).

Unlike the changes identified at Env375, which have not been reported to naturally occur in SHIV infected rhesus macaques, mutations at Env281 were discovered following passage of several SHIV clones *in vivo* [38]. It was shown that like the Env375 mutations, these Env281 mutations could increase infectivity in association with increased binding of HIV-1 Env to rhesus CD4. We generated Env281 mutations alone and in combination with Env375 mutants to look for additive, synergistic, or antagonistic effects on replication in primary rhesus CD4^+^ T cells. We selected the 4 SHIVs with the best replicative potential (CAP220_766_, CAP224_766_, CAP304_766_, and CAP174_766_) to assess the impact of mutations at site 281 on replication. Because these clones carried polymorphisms at 281 (CAP220-V, CAP224-A, CAP304-S, and CAP174-S), 4 SHIV subclones were made containing either serine, alanine, valine, or threonine. Three mutant clones had insufficient infectivity based on TZM-bl assay for use in replication assay (CAP220_766_281T and -281S and CAP304_766_281V). Clones CAP224_766_281S, CAP304_766_281T, CAP174_766_281T and CAP174_766_281V replicated well on rhesus CD4^+^ T cells (Fig 5 dashed lines). Surprisingly neither CAP224_766_281V nor -281T replicated well despite high infectivity titers on TZM-bl cells, which express human CD4. We assessed if combined mutations at both residues 281 (S, A, V, and T) and 375 (CAP224_766_375W, CAP304_766_375H, and CAP174_766_375H) would reveal an incremental replication benefit (Fig 5 solid lines). Combining the two mutations conferred no additional benefit in replication over the single mutations except that the 375-mutation rescued Env281 mutant clones that failed to replicate well alone. Overall, either 281 or 375 mutations appear to increase suboptimal replication and these mutations do not exhibit incremental effects in combination.

### Antibody neutralization

**Fig 6:**
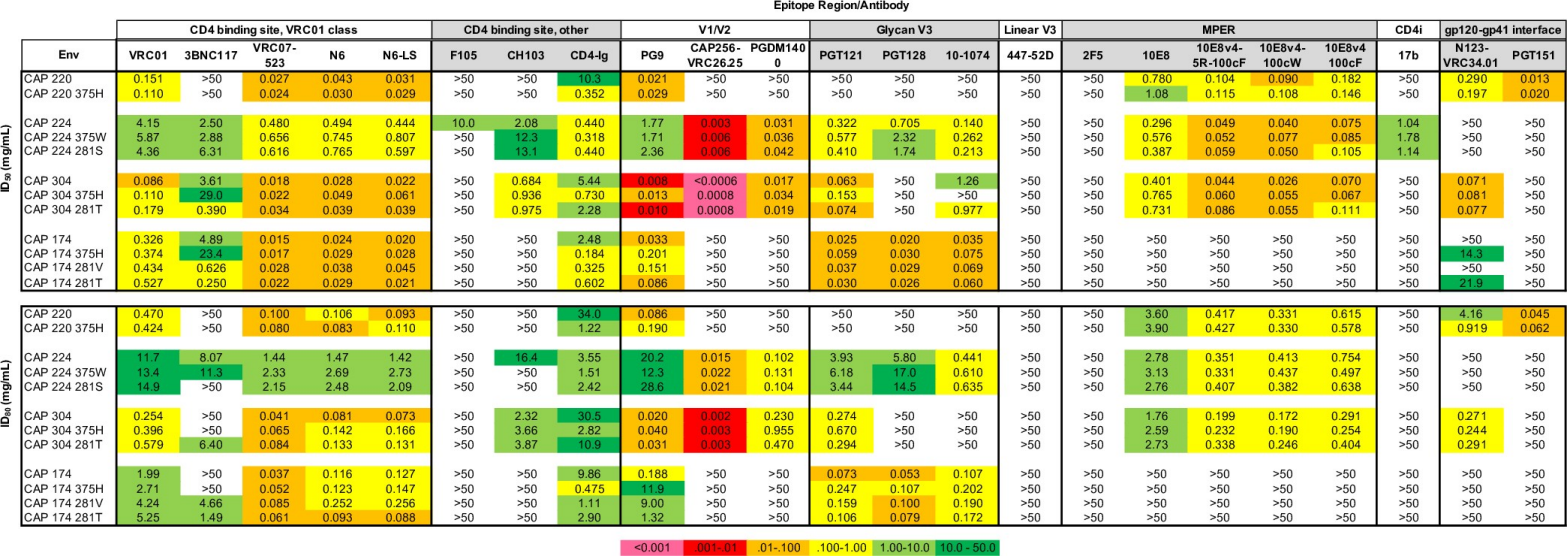
Neutralization sensitivity of HIV Env and SHIVs with 281/375 mutations. Neutralization sensitivity is reported as the concentration (μg/mL) at which relative luminescence units were reduced 50% (ID_50_) or 80% (ID_80_) compared to virus control wells.

To assess the neutralization profile of the replication competent SHIVs we constructed and whether mutations at 281 or 375 resulted in alterations in Env neutralization phenotype, we compared antibody neutralization sensitivities of selected SHIVs using a panel of well characterized HIV-1 mAbs (Fig 6). Our panel comprised antibodies targeting the CD4 binding site (VRC01, 3BNC117, VRC07-523-LS, N6, N6-LS, F105, CH103, and CD4-Ig), V1/V2 (PG9, CAP256-VRC26.25, and PGDM1400), V3 glycan (PGT121, PGT128, and 10–1074), linear V3 (447-52D), MPER (2F5, 10E8, 10E8v4-5R-100cF, 10E8v4-100cW and 10E8v4-100cF [49]), CD4i (17b), and the gp120-gp41 interface (N123-VRC34.01 and PGT151). We assessed neutralization profiles of replication competent SHIVs by determining the 50% and 80% inhibitory concentration (ID_50_ and ID_80_) of the full-length SHIV to account for the HIV gp41 interactions with SIV Gag matrix that might alter trimer conformation and neutralization profiles [31]. All parental SHIVs were resistant to 447-52D, 2F5, 17b (except CAP224), and F105 except (CAP224). CAP220 was resistant to all monoclonal antibodies (mAbs) targeting V3 glycan epitopes, CAP174 was resistant to mAbs targeting MPER, and CAP224 was resistant to mAbs targeting the gp120-gp41 interface. All showed sensitivity to mAbs of VRC01 class targeting the CD4 binding site, with CAP224 being the most resistant to neutralization at this epitope. CAP220 and CAP174 exhibited strong resistance to neutralization at V1/V2 loop epitopes. CAP220, CAP304, and CAP174 show neutralization profiles consistent with tier 2 viruses [50]. Due to its sensitivity to the CD4-induced epitope antibody, 17b, CAP224 can be classified as a tier 1, neutralization sensitive, virus. The neutralization profiles for both the selected 375 and 281 variants mirror their parental profiles. Sensitivity to those antibodies targeting the CD4 binding site were exceptions, though not surprising since the mutations affect this region, increasing the affinity of the HIV gp120 for rhesus CD4. Upon mutation at 281 or 375, CAP220, CAP304, and CAP174 became more sensitive to CD4-Ig, a trend consistent with findings from Li *et al* [31]. CAP304 and CAP174 became more resistant to 3BNC117 with the 375H mutation and more sensitive with the 281V/T mutation. While CAP224 was the only Env sensitive to F105 and CH103, all of its 281 and 375 variants showed higher resistance to neutralization by these antibodies. CAP304 became resistant to neutralization by 10–1074 with the 375H mutation. CAP174 became modestly more resistant to PG9 with 375H and 281V mutations and slightly more sensitive to NR3-VRC34.01 with 375H and 281T mutations. Overall, these selected 281 and 375 mutations generated only modest changes to antibody neutralization profiles, and presumably Env trimer structure, but substantially increased viral replication *in vitro* and *in vivo*.

We examined the plasma of animals in the SHIV Env375 mutant cohort for the development of autologous neutralizing antibodies at 5 and 21 wpi (S3 Fig). Plasma was neutralizing only to the virus with which the animal was inoculated, with the possible exception of animal N037 showing some cross-reactivity with CAP224 375W at 21 wpi. Neutralization was generally weak at 5 wpi and potency increased at 21 wpi particularly in animals E101, V095, V073, and V092. Those animals with higher peak viremia and higher viral load during the chronic phase of infection had better neutralizing plasma, likely related to increased availability of antigen. Since viral persistence and autologous neutralizing antibodies were inversely correlated, other factors likely contribute to the typically low viral persistence that we observed.

### Co-receptor usage of modified SHIV

**Fig 7:**
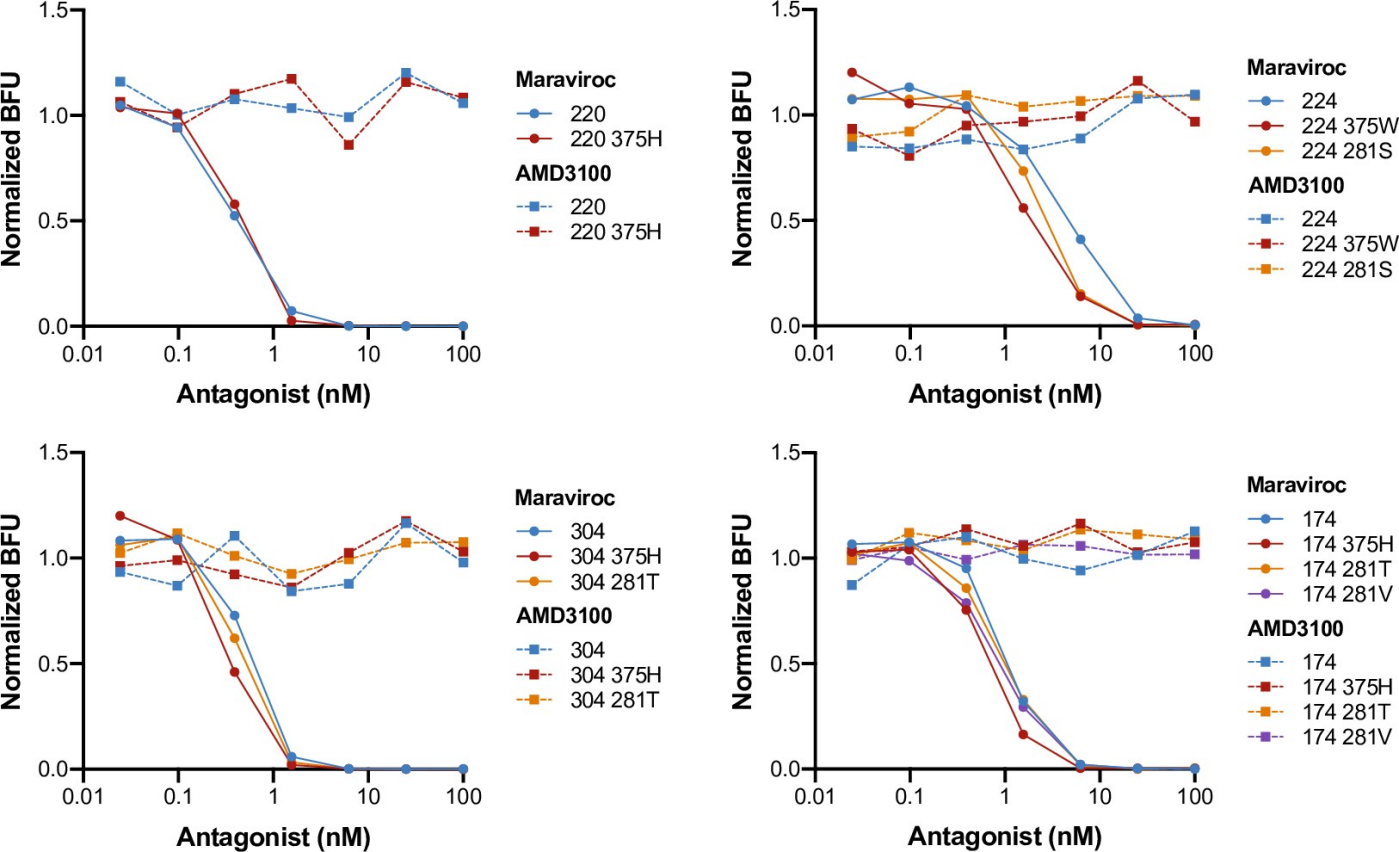
Co-receptor usage of SHIV with 281/375 mutations. Co-receptor usage was assessed for parental and Env281/375 mutant SHIV using a viral entry inhibition assay. Maraviroc, a CCR5 antagonist, and AMD3100, a CXCR4 antagonist, were used in conjunction with TZM-bl reporter cells, which contain a Tat-inducible β-galactosidase gene expression cassette. Blue forming units (BFU) corresponding to infected cells are reported normalized to those with culture lacking Maraviroc and AMD3100.

One potential concern with *in vivo* passage to improve SHIV replication is a change in viral tropism. To confirm that that the engineered changes at Env375 and Env281 in CAP220, CAP224, CAP304, and CAP174 do not lead to alteration in co-receptor usage, we employed a viral entry inhibition assay using Maraviroc (CCR5 antagonist) and AMD3100 (CXCR4 antagonist). AMD3100 had no effect on viral entry for the parental SHIV as expected [48] nor on any of the mutant SHIVs (Fig 7). Conversely, when Maraviroc was present, viral entry for parental and mutant SHIVs exhibited a dose-inhibition response. Thus, Env375 and Env281 mutation did not alter co-receptor usage in our SHIVs and they remained exclusively CCR5 tropic viruses.

## Discussion

SHIV infection of macaques currently provides the best animal model for preclinical evaluation of HIV Env targeted vaccines and passive antibody interventions, especially for prophylactic approaches. However, one critical problem using SHIVs for evaluating post-infection therapeutic regimens is that they most often replicate to lower levels in macaques than wild-type SIV or HIV-1 in humans. Historically, SHIV replication *in vivo* was augmented by serial *in vivo* passage in macaques but doing so was associated with sometimes catastrophic consequences: CXCR4 co-receptor tropism switch [27] or an altered neutralization profile [27, 34, 37, 40–42]. Recently, two studies have described screening approaches and a rational design strategy to select Envs that increase SHIV *in vivo* replication without adverse effects [31, 38]. Here we expanded on these studies by generating and characterizing 19 globally relevant subtype C SHIV clones using similar strategies. We evaluated their capacity for replication in rhesus macaque primary CD4^+^ T cells and *in vivo* in rhesus macaques. Following a pooled inoculum *in vivo* competition screen, we found 7 SHIVs that were detectable in at least one animal. Of these, we chose 3 associated with the highest levels of peak viremia and 3 that were not detectable in any animal and mutated these viruses at Env positions 281 and 375. The Env375 mutations dramatically improved *in vitro* replication and in animals containing a pool of all 375 mutations, peak viremia increased in magnitude and arrived at peak 1–2 weeks earlier than animals infected with the matched parental viruses. These results suggest that Env375 mutations not only augment *in vivo* replication of SHIV that show some competence for replication but can also enhance replication for some SHIVs with minimal capacity to replicate in macaque cells. Additionally, CD4^+^ T cell depletion was observed in the context of 375 mutation but not during the competition screen. Since many SHIVs do not replicate well, this rational alteration could streamline the time- and resource-intensive development of SHIVs. Env375 mutation only appeared to impact acute phase viral dynamics with little consistent improvement in setpoint viremia. That the mode of augmented viral replication during acute infection has little impact during chronic infection is both fascinating and unexpected and a topic for further research to increase the utility of SHIVs for therapeutic studies.

In subtype C clones, histidine, tryptophan, tyrosine, or phenylalanine substitutions at Env375 provided a selective advantage over methionine and serine *in vitro* and *in vivo*. At peak viremia and chronic infection, clones with histidine at this residue were most commonly found but not frequently enough to conclude that 375 His is the preferred allele for subtype C viruses generally. In fact, in other studies of SHIVs containing other subtype C primary Envs, His, Phe, Tyr and Trp were each preferred in particular Env backgrounds for efficient replication in rhesus animals [31]. As a practical matter, once a primary HIV-1 Env is cloned as a SHIV, it is technically straightforward to exchange 375 codons to represent the six alleles, which can be tested as an inoculum mixture in a few macaques to quickly identify preferred alleles for individual clones [31]. This is a small investment of time and resources given the paramount importance of maximizing replication fitness of novel SHIVs. Furthermore, while mutations in residue 281 naturally occur in SHIVs during animal passage, we found that mutations at this residue can augment SHIV replication *in vitro*, but not all mutations were successful, and these differences appear to be context dependent. SHIV with both 281 and 375 mutations did not exhibit any incremental benefit *in vitro*. Overall, the context of the backbone envelope affects the efficiency of 281 and 375 mutations to enhance rhesus CD4 binding and increase viral replication with preferential mutations in SHIV C of 281S, V and T, and 375H and W.

To place these results into a structural context, we analyzed the location of residues 281 and 375 in the context of the CD4-bound conformation of the HIV-1 Env trimer [51](S4A and S4B Fig). Residues 281 and 375 were distal from each other and interacted with different regions of CD4. Residue 281 is located within the footprint of CD4 and mutating from alanine to serine is predicted to increase intermolecular hydrogen bonding (S4C and S4D Fig). Residue 375 is mostly occluded from the surface of gp120 (S4E and S4F Fig), being buried within an interfacial pocket [52], and likely exerts its effects through conformational effects, not direct interaction with CD4. The structural analysis thus clarifies that the lack of synergy of the double mutation is not due to overlapping sites of interaction.

Importantly, the 4 SHIVs we developed here have heterogeneous neutralization sensitivity profiles that may be beneficial in therapeutic development or testing various passive administered antibodies to block infection. Antibody neutralization sensitivity for the selected Env375 and Env281 variants mostly matched that of the corresponding parental viruses with occasional inconsistencies, generally including those antibodies with epitopes in the CD4 binding site. This observation is not surprising since the Env281 and Env375 mutations alter CD4 binding affinity. There is little evidence for gross quaternary conformational changes in these modified SHIV clones exist as can happen with adapted SHIV [43]. However, alterations in sensitivity do suggest slight changes in the accessibility of some epitopes, particularly in the CD4 binding site. Importantly, these mutations did not affect co-receptor usage and the mutant SHIVs remained CCR5 tropic. Overall, these mutations augment replication without significantly altering phenotype and the SHIV development approach described here should be useful for the derivation of SHIVs from other globally relevant virus strains.

## Materials and methods

### Ethics statement

18 purpose-bred Indian-origin rhesus macaques were housed and cared for in accordance with local, state, federal, and institute policies in an American Association for Accreditation of Laboratory Animal Care-accredited facility at the National Institutes of Health (NIH) or at a contract facility (Bioqual Inc., Rockville, MD). All animal experiments were reviewed and approved by the Animal Care and Use Committee of the Vaccine Research Center, NIAID, NIH and covered under protocol VRC 14–494 and adhered to the standards of the NIH Guide for the Care and Use of Laboratory Animals and the Animal Welfare Act.

### Plasmid construction

20 T/F viral subtype C *env* clones derived from acutely HIV-1 infected individuals in the CAPRISA cohort in South Africa were cloned into the pKB9 [37] and pSHIV.D.191859.dCT [31] vectors. For pKB9, PCR amplification of *env* utilized primers (5’-TATGGGGTACCGGTGTGG-3’) and (5’-GCGATGGGTCTCCTCGAGGTTGGGAAGCGGGTCTG-3’). Amplicons were ligated with pKB9 using *Age*1 and *Xho*1. Because CAP177 and CAP206 contained an internal *Xho*1 site, *Bsa*1 was used to create an *Xho*1 overhang. To facilitate cloning into pSHIV.D.191859.dCT, a *BsmB*1-*Age*1-*BsmB*1 sequence (5’-TGAGACGACCGGTCGTCTCA-3’) replaced *env* between the signal peptide and *tat*-*rev* splice site to create pSHIV.D.entry. Sequences for *env* were PCR amplified using (5’-GCGATGGCGGCCGCCGTCTCACACAGTTTATTATGGGGTACCTGTG-3’) and (5’-GCGATGCGTCTCAGGTGAGTATCCCTGCCTAA-3’) with the exception of CAP332 for which (5’-GCGATGCGTCTCAGGTGAGTAACCCTGCCTAA-3’) was used. Amplicons were ligated with pSHIV.D.entry using *BsmB*1. To introduce 375 mutations in CAP220, CAP224, CAP304, CAP200, CAP174, and CAP382, the previous *env* amplicons were generated in two halves with internal *BsmB*1 sites introduced during PCR amplification. Likewise, to introduce 281 mutations in CAP220, CAP224, CAP304, CAP174, CAP220 375H, CAP224 375W, CAP304 375H, and CAP174 375H, *env* amplicons were generated in two halves with internal *BsmB*1 sites introduced during PCR amplification. The overhangs generated upon restriction digest bore the appropriate mutations and formed a seamless sequence when the two halves were ligated with pSHIV.D.entry using *BsmB*1. New viral clones and mutants were sequenced to confirm accurate plasmid construction.

### Virus production

Virus was prepared in HEK-293T cells (ThermoFisher) transfected with the appropriate SHIV plasmid using Mirus Trans-IT 293 transfection reagent as described by the manufacturer. Cells (2×10^6^) were plated in 5 mL D10 (DMEM with 10% FBS (Atlanta Biologicals), 2 mM L-glutamine, and 100 U/mL penicillin-100 μg/mL streptomycin) 24 hr prior to transfection. Culture medium was doubled at 24 hr post-transfection, and culture supernatants were collected at 48 hr. Supernatants were clarified by centrifugation and 1 mL aliquots of supernatant were stored at −80°C. Viral infectivity was determined using TZM-bl reporter cells (reference no. 8129; NIH AIDS Research and Reference Reagent Program), which contain a Tat-inducible luciferase and β-galactosidase gene expression cassette, as previously described [53].

### *In vitro* replication

Rhesus macaque peripheral blood mononuclear cells were isolated from whole blood using SepMate tubes (StemCell Technologies) with Lymphoprep (StemCell Technologies) density gradient medium and centrifugation. CD4^+^ T cells were enriched by negative selection (CD4 T Cell Isolation Kit, Miltenyi). Enriched rhesus CD4+ T cells were activated with αCD2/αCD3/αCD28 beads (T Cell Activation Kit, Miltenyi) per the manufacturer’s instructions and cultured with 100 U/mL IL-2 in RPMI medium supplemented with 10% FBS, 1% L-glutamine, and 1% penicillin/streptomycin (RPMI Complete) at a density of 2-3×10^6^ cells/mL. After 72 hr activation beads were removed by magnet, according to kit instructions. Cells were maintained in RPMI Complete with 100 U/mL IL-2 for the duration of the experiment. For each experiment, individual SHIVs were used to infect 1×10^6^ activated rhesus CD4^+^ T cells from three naïve animals mixed equally prior to use. Using a MOI of 0.01–0.02 in a total volume of 1.5 mL, infections were performed by spinoculation at 800×*g* for 2 hours at room temperature, then incubated at 37°C for 2 hr. At the end of this incubation period, cultures were washed 3 times (5–10 mL) in RPMI Complete to remove excess virus, then incubated at 37°C. Culture supernatants were collected over 14 days, with media replacement at each collection time point to maintain cultures in 2 mL total volume. Viral p27 protein was quantified by ELISA (Advanced Bioscience Laboratories) with a limit of detection of 62.5 pg/mL.

### Viral entry inhibition assay

TZM-bl reporter cells were plated at 10^4^ cells per well in 96 well plates (Costar 3917) and allowed to incubate overnight. Cells were incubated with Maraviroc (Selleckchem S2003) and AMD3100 (Selleckchem S8030) for one hour. Concentrations began at 200 nM and were diluted serially 4-fold. Six hundred infectious units of virus stock with 400 μg/mL Polybrene were added at equal volumes thus diluting the antagonist concentration and polybrene 2-fold. Plates were allowed to incubate for 48 h. Samples were then processed as previously described [53].

### Animal experiments

At the start of the study, all animals were free of cercopithecine herpesvirus 1, simian immunodeficiency virus (SIV), simian type-D retrovirus, and simian T-lymphotropic virus type 1. All animals were treated with enrofloxacin (10 mg/kg once daily for 10 days), paromomycin (25 mg/kg twice daily for 10 days), and fenbendazole (50 mg/kg once daily for 5 days) followed by weekly fecal culture and parasite exams for 3 weeks to ensure they were free of common enteric pathogens. At least a 4-week post-treatment period allowed time for stabilization of the microbiome prior to use in this study.

For the initial screening of the 19 Env variants, SHIV clones were divided into 9 pools with 6 variants in each pool. Each variant was represented in 3 pools, and pairs did not occur more than twice (Fig 2A). Variants comprised the SIVmac239 and SIVmac766 backbone equally. CAP206 and CAP330 were exceptions: they were treated as a pair as each Env was in only one backbone (SIVmac293 and SIVmac766 respectively). Nine rhesus macaques were each inoculated with one SHIV pool at a dose of 1x10^5^ IU for each variant (as measured on TZM-bl). For the Env375 mutation study, pairs of rhesus macaques were each inoculated with pooled variants of CAP220, CAP224, or CAP304 each containing the full complement of 375 mutations (S, M, Y, H, W, and F) at a dose of 1x10^4^ IU for each variant. All 6 mutations at 375 in 3 separate clones (CAP174, CAP200, and CAP382) were pooled to infect three rhesus macaques at 1x10^4^ IU for each variant. Inoculations were performed intravenously.

Whole blood was collected from sedated animals. Plasma for viral RNA quantification and sequencing analysis and peripheral blood mononuclear cells (PBMCs) for sequencing analysis were prepared from blood collected in EDTA anticoagulated Vacutainer tubes (BD). Following separation from whole blood by centrifugation, plasma aliquots were stored at -80°C. PBMCs were isolated from whole blood by Ficoll-Paque Plus (GE Healthcare) gradient centrifugation; serum was prepared from serum collection tubes.

### Neutralization tier phenotyping and neutralization assays

Neutralization of replication-competent SHIVs was evaluated *in vitro* using TZM-bl target cells and a luciferase reporter assay as described [54–56]. Briefly, the SHIVs were generated by transfection in 293T cells. Viruses were incubated with antibody or plasma for 30 min at 37°C before TZM-bl cells were added. The HIV protease inhibitor indinavir was added to wells at a final concentration of 1 μM to limit infection of target cells to a single round of viral replication. Luciferase expression was quantified 48 hr after infection upon cell lysis and the addition of luciferin substrate (Promega).

### Viral load measurements

Virions were pelleted from plasma and virion-associated RNA extracted as previously described [31]. Briefly, SHIV plasma viral load determinations over the duration of the study were quantified using a two-step real-time qRT-PCR based on amplification of an SIVmac239-derived sequence located in the Gag coding region. As used in the present study, the limit of detection for this assay is 15 vRNA copies/mL.

### Single-genome amplification/sequencing of SHIV *env*

To generate *env* cDNA, reverse transcription of viral RNA was performed using SuperScript III reverse transcriptase according to the manufacturer’s directions (Invitrogen) and a gene specific primer (5’-TGTAATAAATCCCTTCCAGTCCCCCC-3’). The *env* gene was then amplified using nested PCR (round 1 (5’-CCTCCCCCTCCAGGACTAGC-3’) and (5’-TGTAATAAATCCCTTCCAGTCCCCCC-3’); round 2 (5’-GACCTCCAGAAAATGAAGGACCAC-3’) and (5’-ATGAGACATRTCTATTGCCAATTTGTA-3’). Nested-PCR amplification was performed using Platinum *Taq* DNA High Fidelity polymerase (Thermo Fisher Scientific) for both reactions according to the manufacturer's protocol. Briefly, 1× High Fidelity Platinum PCR buffer, 2 mM MgSO_4_, 0.2 mM each deoxynucleoside triphosphate, 0.2 μM each primer, and 0.025 U/μl Platinum *Taq* High Fidelity polymerase were combined in a 10 μl reaction mixture. First-round PCR mixtures were denatured at 94°C for 2 min; followed by 35 cycles of 94°C for 15 sec, 55°C for 30 sec, and 68°C for 4 min; and terminated with a single 15 min 68°C extension. Template cDNA was serially diluted until PCR-positive wells, scored based on gel electrophoresis, constituted less than 30% of the total number of reactions, as previously described [57, 58]. Next, 1 μl of each reaction mixture was transferred to a second-round reaction, which was amplified under the same PCR conditions but for 45 cycles. Correctly sized amplicons were sequenced directly using Big Dye Terminator technology (Applied Biosystems). Both DNA strands were sequenced and overlapping sequence fragments for each amplicon were assembled and edited using the Sequencher 5.0 program (Gene Codes). To confirm PCR amplification from a single template, chromatograms were manually examined for multiple peaks. Sequences with mixed bases were excluded from further analysis. The remaining sequences were used to assess the frequency and number of *env* sequences to determine proportionality [57].

### 375 proportion determination using MiSeq

RNA was isolated from plasma using QIAamp Viral RNA mini kit (Qiagen) per the manufacturer’s instructions and synthesized into cDNA using SuperScript III reverse transcriptase (Invitrogen) with reverse primer (5’-ATGGGAGGGGCATACATTGC-3’) for the 375 mutation. qPCR was used to quantify the cDNA synthesized. Amplicons of the cDNA with MiSeq adaptors appended were generated using PCR amplification with Platinum *Taq* High Fidelity. For template, 5×10^3^ copies to 1×10^6^ copies of cDNA were used per reaction. First-round PCR mixtures were denatured at 94°C for 2 min; followed by 40 cycles of 94°C for 15 sec, 60°C for 90 sec, and 68°C for 30 sec; and terminated with a single 5 min 68°C extension. The annealing sequence for the P5 MiSeq primers was (5’-GAGGGGAYCTAGAARTTACAACAC-3’), but CAP382 required a non-degenerate annealing sequence (5’-GAGGGGATCTAGAAATTACCACAC-3’). The MiSeq primers were used in conjunction with the P7 MiSeq primer (5’-TGGGAGGGGCATACATTGC-3’) for amplification.

To process this sample library, 10 μL from each reaction were pooled, and purified using the QIAquick PCR purification kit (Qiagen). The DNA was quantified using the QuBit and diluted to 3.0 nM. From the diluted sample, 5 μL were placed in a new tube and denatured with 5 μL of 0.2 M NaOH before vortex and centrifugation at 280×g for 1 min. Following incubation at room temperature for 5 min, 990 μL of chilled HT1 buffer was added. This solution was subsequently diluted to 12.5 pM. The control PhiX library was treated similarly. From the processed sample library, 2 μL were combined with 3 μL of a Tris-HCl pH 8.5, 0.1% Tween-20 solution. Subsequently, 5 μL of 0.2 M HCl was added, and the sample vortexed and centrifuged at 280×g for 1 minute. The sample was incubated at room temperature for 5 min, and 990 μL of chilled HT1 buffer added. Multiplexed samples and PhiX library were then loaded on the MiSeq reagent tray, and the run initiated. The proportion of each variant was based on the number of sequence-reads per sample.

### Flow cytometry

Antibodies and reagents were obtained from BD Biosciences, unless indicated otherwise, and data analysis was performed using FCS Express (De Novo Software). Samples were prepared and absolute cell counting and lymphocyte immunophenotyping were performed as previously described [59–61].

## Supporting information

S1 Fig Phylogenetic analysis of SHIV Env clones. 500 HIV-1 subtype C env sequences and subtype reference sequences from the LANL HIV database were aligned to SHIV reported here. Phylogenetic analysis was performed using neighbor joining method. All subtypes segregate accurately, and new clones are found throughout the subtype C phylogeny.
(TIF)

S2 Fig CD4^+^ T cell counts for SHIV infected rhesus macaques. Total, naïve, central memory, and effector memory CD4^+^ T cell counts were determined by flow cytometry for macaques infected with combinatorial pools of SHIV (A) and 375 mutant pools (B). Counts were monitored during the course of infection. The dashed gray box/line indicates the period of peak infection.
(TIF)

S3 Fig Neutralizing antibody sensitivity of SHIV infected macaques. SHIV infected NHP plasma neutralization titers are reported as the reciprocal plasma dilution required to achieve 50% (ID_50_) or 80% (ID_80_) neutralization of autologous and heterologous SHIV.
(TIF)

S4 Fig Structural analysis of residues 281 and 375. (A) The structure of the CD4-bound HIV-1 SOSIP trimer (PDB 5VN3) is shown in cartoon representation with residues 281 (blue) and 375 (magenta) highlighted as spheres. (B) A magnified view of the relevant residues shown as sticks and colored as in panel A. Phe43 is shown in yellow stick representation. (C) The interface between CD4 and gp120 in the region of residue 281 is shown with native alanine. (D) Mutation to a serine or threonine at 281 would provide additional polar contacts to this region. (E) The Phe43 cavity with Ser375 is displayed with a slice through the surface. Of note, this enlarged cavity often contains ordered solvent in high resolution crystal structures. (F) Trp375 fills the cavity while leaving room for CD4 Phe43.
(TIF)
