# Supplementary figures and images for: Rational design and in vivo selection of SHIVs encoding transmitted/founder subtype C HIV-1 envelopes
Source: PLoS Pathog. 2019 Apr 3;15(4):e1007632. doi: 10.1371/journal.ppat.1007632 (PMC6447185; doi:10.1371/journal.ppat.1007632)

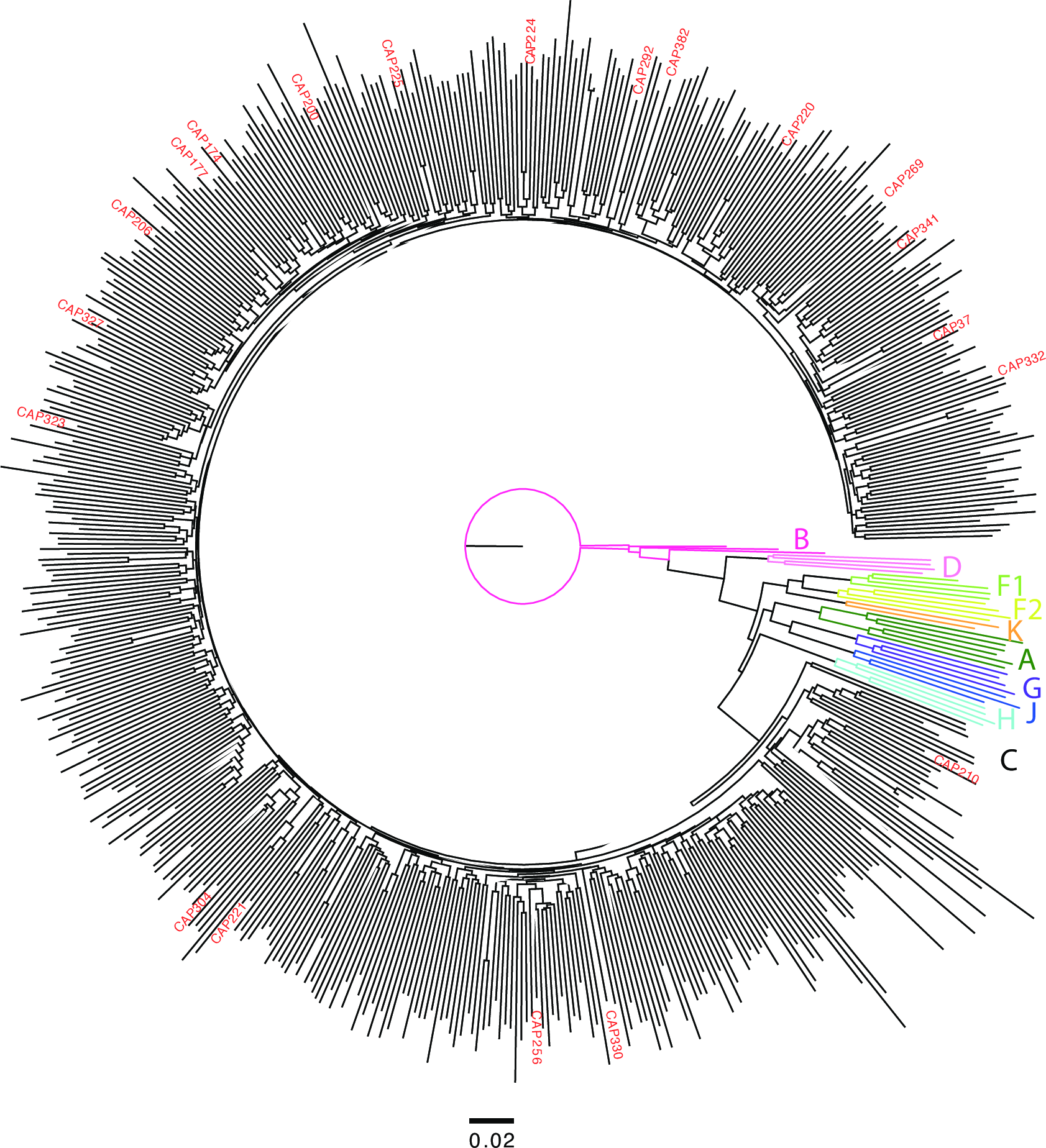

Supplement: S1 Fig — 500 HIV-1 subtype C env sequences and subtype reference sequences from the LANL HIV database were aligned to SHIV reported here. Phylogenetic analysis was performed using neighbor joining method. All subtypes segregate accurately, and new clones are found throughout the subtype C phylogeny. (TIF) [file ppat.1007632.s001.tif]

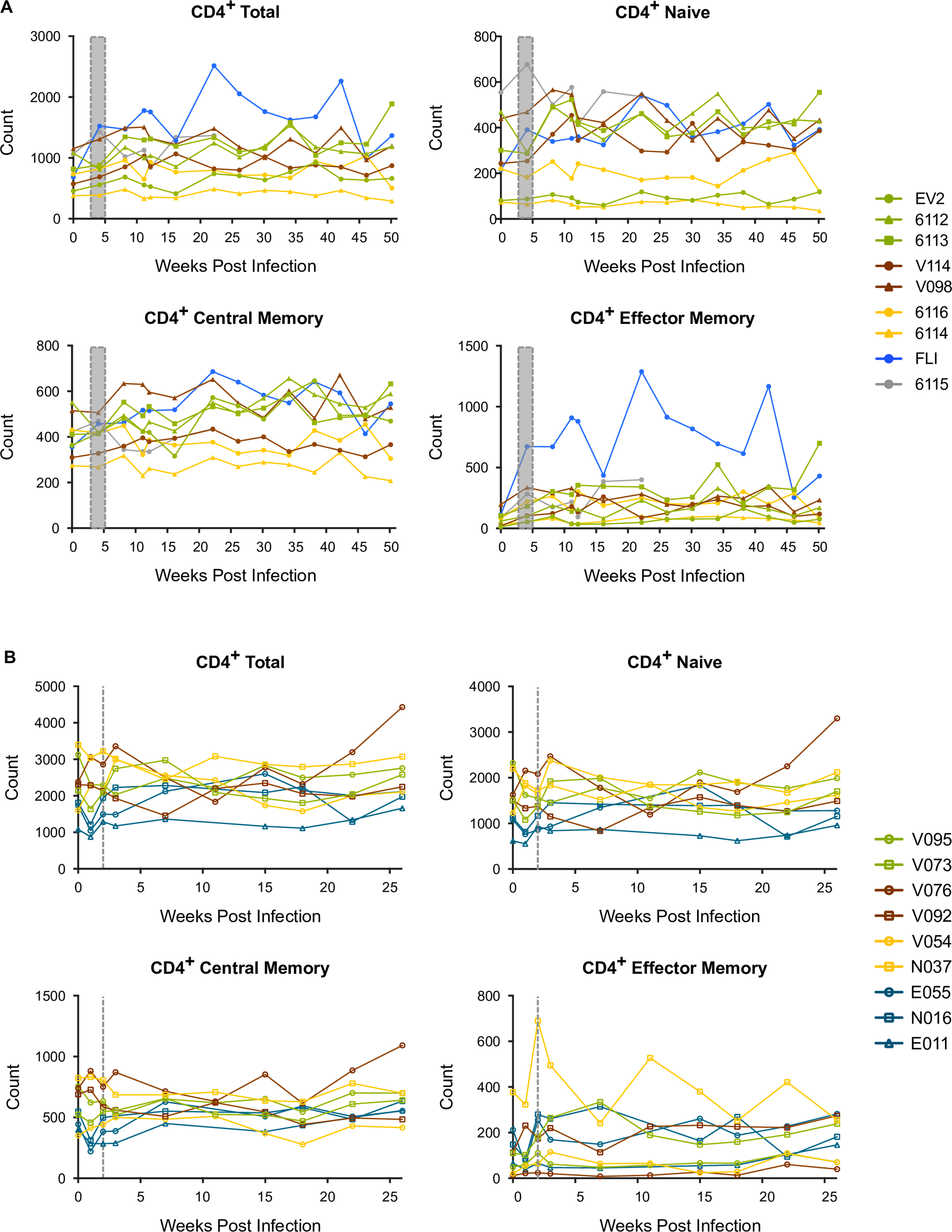

Supplement: S2 Fig — Total, naïve, central memory, and effector memory CD4+ T cell counts were determined by flow cytometry for macaques infected with combinatorial pools of SHIV (A) and 375 mutant pools (B). Counts were monitored during the course of infection. The dashed gray box/line indicates the period of peak infection. (TIF) [file ppat.1007632.s002.tif]

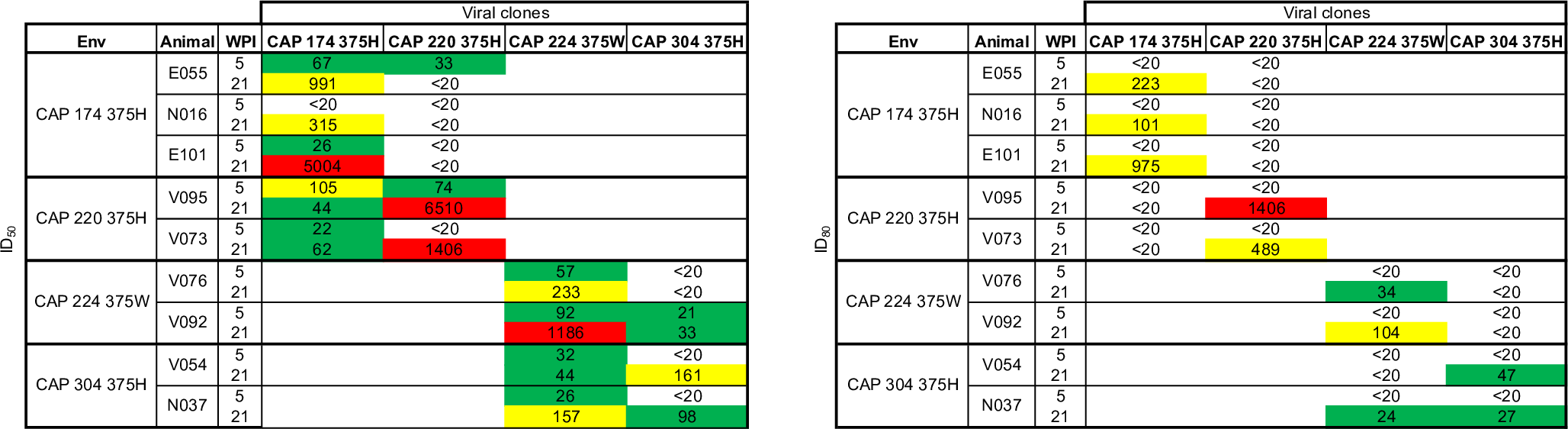

Supplement: S3 Fig — SHIV infected NHP plasma neutralization titers are reported as the reciprocal plasma dilution required to achieve 50% (ID50) or 80% (ID80) neutralization of autologous and heterologous SHIV. (TIF) [file ppat.1007632.s003.tif]

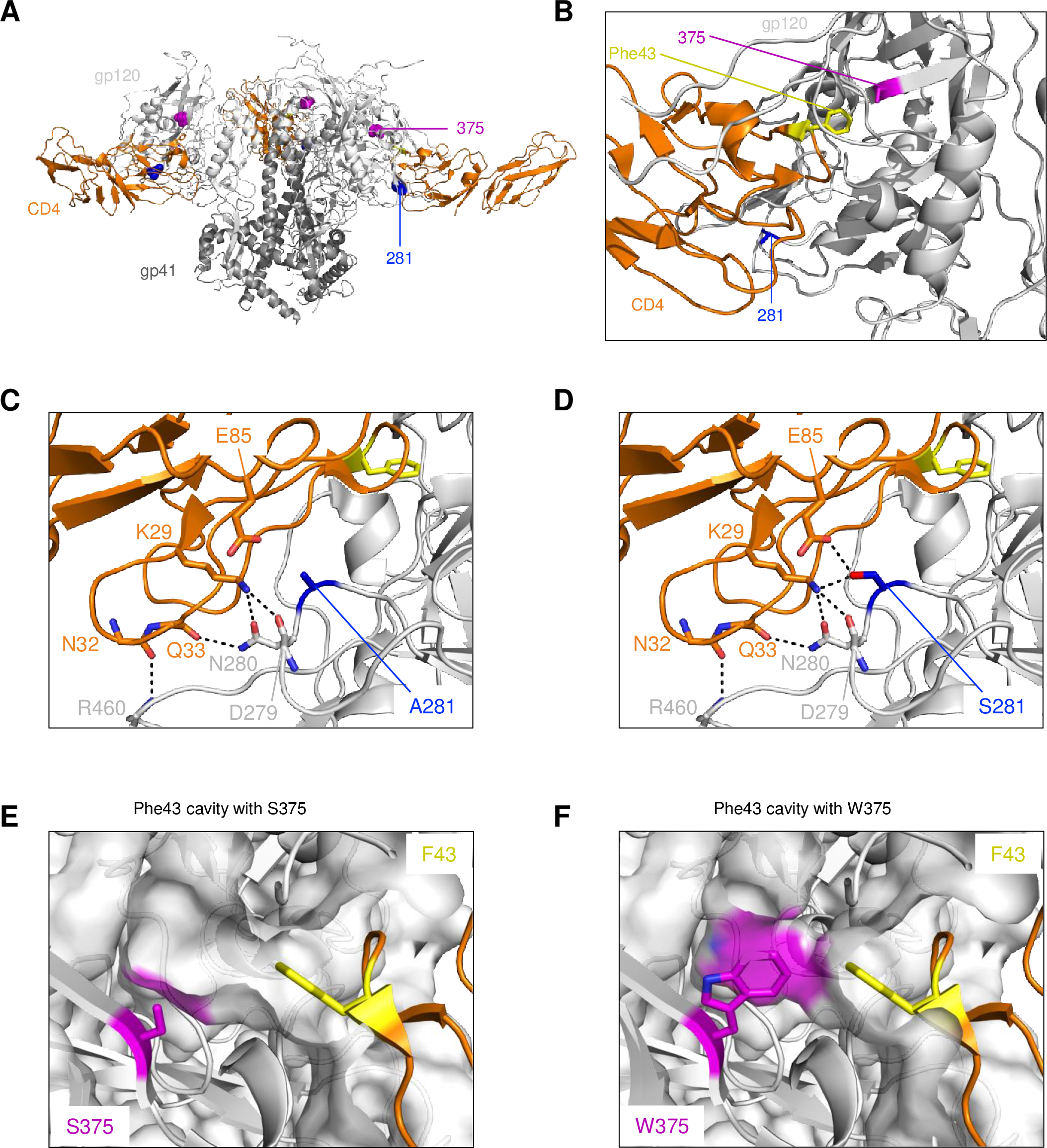

Supplement: S4 Fig — (A) The structure of the CD4-bound HIV-1 SOSIP trimer (PDB 5VN3) is shown in cartoon representation with residues 281 (blue) and 375 (magenta) highlighted as spheres. (B) A magnified view of the relevant residues shown as sticks and colored as in panel A. Phe43 is shown in yellow stick representation. (C) The interface between CD4 and gp120 in the region of residue 281 is shown with native alanine. (D) Mutation to a serine or threonine at 281 would provide additional polar contacts to this region. (E) The Phe43 cavity with Ser375 is displayed with a slice through the surface. Of note, this enlarged cavity often contains ordered solvent in high resolution crystal structures. (F) Trp375 fills the cavity while leaving room for CD4 Phe43. (TIF) [file ppat.1007632.s004.tif]
